# Acute Ischemia of the Left Lower Limb Revealing Infective Endocarditis on a Native Mitral Valve: A Case Report

**DOI:** 10.7759/cureus.108293

**Published:** 2026-05-05

**Authors:** Sidi Mohamed Limame, Taleb Ekhyar Arguiena, Mohamed Issa Elkharchi, Soueid Ahmed Sidi Mhamed, Mohamed Lemine Sidi Oumar

**Affiliations:** 1 Cardiology, Centre National de Cardiologie, Nouakchott, MRT; 2 Cardiothoracic Surgery, Centre National de Cardiologie, Nouakchott, MRT; 3 Faculty of Medicine, Pharmacy, and Odonto-Stomatology, Centre National de Cardiologie, Nouakchott, MRT; 4 Pulmonology, Centre Hospitalier National, Nouakchott, MRT

**Keywords:** acute lower limb ischemia, conservative surgical management, extracardiac complications of infective endocarditis, infective endocarditis, native mitral valve

## Abstract

Infective endocarditis is a serious disease characterized by ulcerative lesions resulting from the implantation of a microorganism on the endocardium. Extracardiac complications of infective endocarditis are often related to the migration of emboli from valvular vegetations. The present report describes an acute left lower limb ischemia revealing infective endocarditis on a native mitral valve in a 29-year-old female. Our case illustrates an unusual clinical presentation of infective endocarditis on a native valve and describes a delayed conservative surgical management of the native valve with a good outcome.

## Introduction

Infective endocarditis is a serious disease with an estimated incidence of 13.8 per 100,000 individuals per year [1] and a hospital mortality rate of around 15% to 20%, despite considerable advances in diagnostic imaging, identification of causative microorganisms, antibiotic therapy, and surgical treatment [2]. It leads to valvular and perivalvular destruction, as well as distal destruction, due to emboli of septic vegetations, metastatic infections, and septicemia. The characteristic lesion of infective endocarditis is a vegetation composed of platelets, fibrin, microorganisms, and inflammatory cells [3]. These complications are frequent, particularly heart failure directly related to valvular damage, valvular abscesses, and systemic embolisms, especially cerebral embolisms, the occurrence of which remains difficult to predict [4]. Streptococci and staphylococci are responsible for approximately 80% of infective endocarditis cases [5]. The diagnosis of infective endocarditis is based on clinical suspicion supported by consistent microbiological data and the documentation of infective endocarditis-related cardiac lesions by imaging techniques [6]. We report the case of a 29-year-old female patient presenting with acute left lower limb ischemia, revealing infective endocarditis of a native mitral valve.

## Case presentation

A 29-year-old female patient with no significant past medical history was admitted to the National Cardiology Center in Nouakchott for acute pain in her left lower limb that had developed suddenly within the past 24 hours, accompanied by a prolonged fever. On admission, her vital signs were as follows: temperature of 38.7°C, heart rate of 120 beats/minute, respiratory rate of 15 breaths/minute, and blood pressure of 120/80 mmHg. Clinical examination revealed a cold and painful left lower limb, with an absent ipsilateral femoral pulse. The patient showed no signs of sensorimotor deficit. Cardiac auscultation revealed a grade 3/6 systolic murmur at the mitral area without a gallop. There were no clinical signs of heart failure. The physical examination, including neurological, pulmonary, and abdominal examinations, was normal. No cutaneous, oral, or urinary tract infection was found.

The electrocardiogram showed regular sinus tachycardia at 118 beats/minute with left ventricular hypertrophy. Laboratory tests revealed microcytic hypochromic anemia, a biological inflammatory syndrome, leukocytosis, and a normal platelet count. Serum creatinine, blood glucose, and electrolytes were normal. Liver function tests were normal (Table 1). Bacteriologically, a single blood culture revealed *Staphylococcus intermedius* sensitive to vancomycin and gentamicin. Urine culture, human immunodeficiency virus serology, and hepatitis virus serology were negative. The chest X-ray was normal.

**Table 1:** Laboratory values ​​of the patient at the time of admission.

Blood tests	Values	Reference range (unit)
Alanine aminotransferase	15	7–56 (U/L)
Aspartate aminotransferase	13	10–40 (U/L)
Alkaline phosphatase	51	44–147 (U/L)
Albumin	4.1	3.5–5.5 (g/dL)
Bilirubin	0.9	0.1–1.2 (mg/dL)
Gamma-glutamyltransferase	17	9–48 (U/L)
Total protein	5	6.0–8.3 (g/dL)
Prothrombin time	10	10.9–12.5 (seconds)
Estimated glomerular filtration rate	107	>90 (mL/minute/1.73m²)
Glycemia	4.1	3.9–5.5 (mmol/L)
Calcemia	8.7	8.5–10.5 (mg/dL)
Vitamin D	37	30–50 (ng/mL)
Hemoglobin	8	13.8–17.2 (g/dL)
Mean corpuscular volume	59	80–100 (fL)
White blood cell count	15,000	4,000–11,000 (µL)
Platelets	300,000	150,000–450,000 (µL)
High-sensitivity troponin	1	<22 (ng/L)
C-reactive protein	186	<1 (mg/dL)
Sodium	142	135–145 (mEq/L)
Potassium	3.7	3.5–5.0 (mEq/L)
Chloride	100	96–106 (mEq/L)

**Figure 1:**
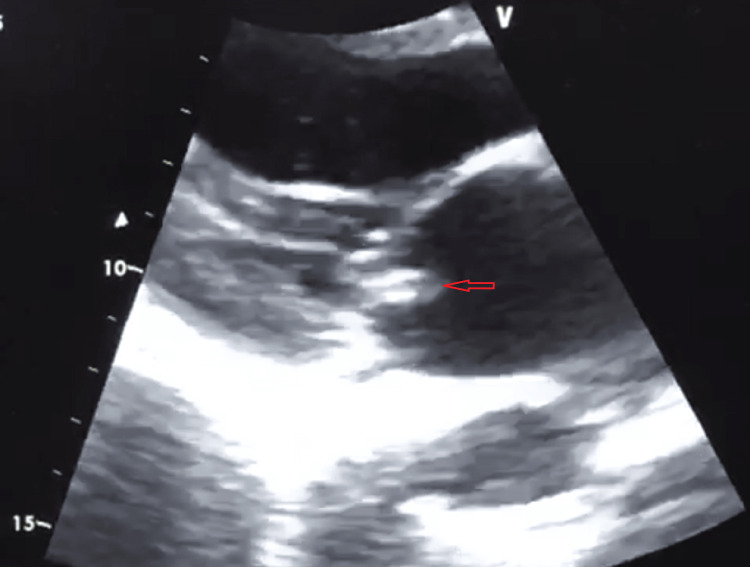
Transthoracic parasternal long-axis view. The image shows the mitral valve apparatus without remodeling or calcifications, with the presence of a hyperechoic mass of irregular consistency, suggestive of vegetation (arrow).

**Figure 2:**
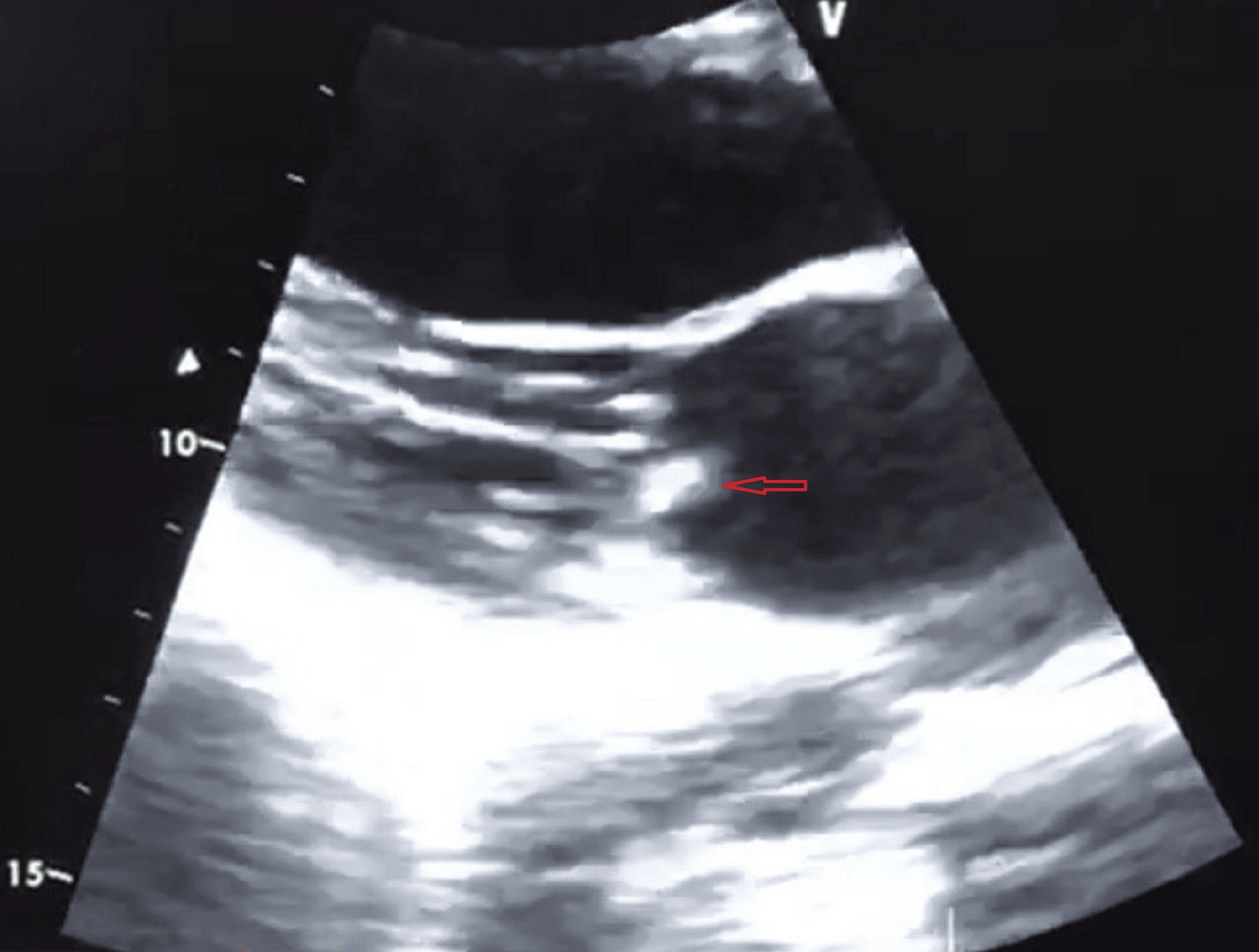
Transthoracic parasternal long-axis view. The image shows vegetations on the auricular side of the small mitral valve (arrow).

Transthoracic Doppler echocardiography revealed mild dilation of the left heart chambers, good systolic function of both ventricles, grade 3 mitral regurgitation with restriction of the anterior leaflet, and a 7 mm rounded image suspended on the atrial side of the anterior mitral leaflet, suggestive of vegetation (Figures 1, 2). The severity of mitral regurgitation was assessed using a multiparameter method. Quantitative analysis by proximal isovelocity surface area demonstrated grade 3 mitral regurgitation with an effective regurgitant orifice area of 0.32 cm² and a regurgitant volume of 49 mL. The other leaflets were normal. Transesophageal echocardiography was performed, confirming the results of the transthoracic echocardiography with the presence of a mobile vegetation. A thoraco-abdominal-pelvic and cerebral CT scan did not reveal any other septic foci.

**Figure 3:**
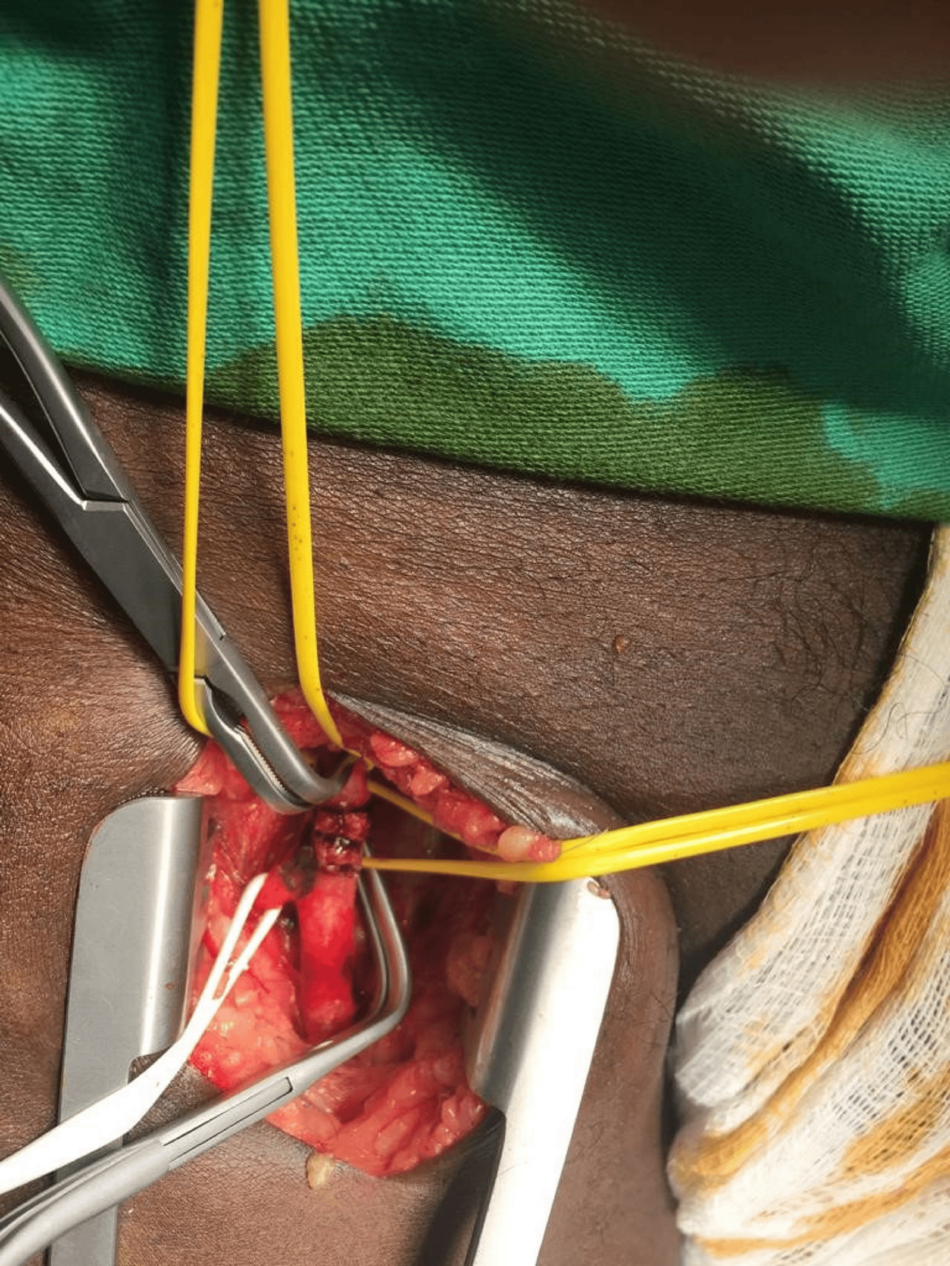
Surgical approach of the left femoral bifurcation embolectomy by Fogarty catheter.

**Figure 4:**
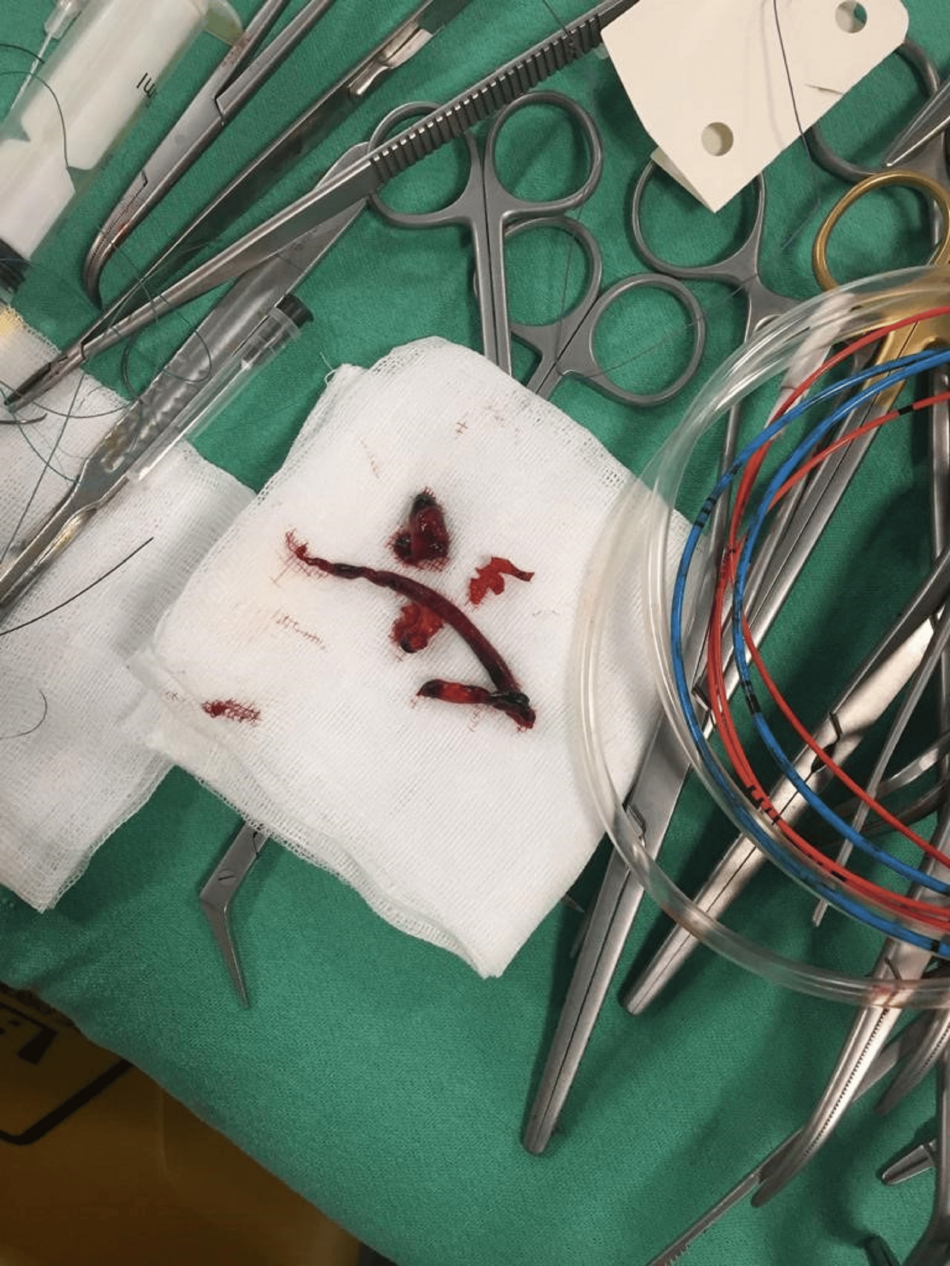
Macroscopic appearance of the embolus extracted by Fogarty catheter.

Given the clinical presentation of acute ischemia of the left lower limb, the patient underwent emergency surgery of common femoral artery embolectomy by Fogarty catheter with a favorable surgical outcome. The nature of the embolus was confirmed by histopathological examination (Figures 3, 4).

The diagnosis of infective endocarditis complicated by acute lower limb ischemia was confirmed according to the modified Duke criteria, with the presence of two major criteria. The patient was started on a four-week course of injectable dual antibiotic therapy with vancomycin and gentamicin. Her hospital stay was favorable, marked by the recovery of femoral, popliteal, posterior tibial, and dorsalis pedis pulses in the left lower limb, resolution of fever by day eight, normalization of the inflammatory markers by day 12, and a negative blood culture. Echocardiography showed the disappearance of the mitral vegetation, but persistence of grade 3 mitral regurgitation. The patient was discharged home after 30 days of hospitalization. The one-year follow-up was marked by worsening mitral regurgitation, which became severe and symptomatic. The patient underwent a planned mitral valve repair with an uncomplicated postoperative course. The outcome at four years of follow-up was favorable with no endocarditis recurrence.

## Discussion

The patient presented with a clinical picture suggestive of infective endocarditis, revealed by acute lower limb ischemia. This endocarditis was likely caused by the pathogen *Staphylococcus intermedius*, documented by a single blood culture with visualization of vegetations on the small mitral valve on transthoracic echocardiography and probable systemic embolization leading to acute left lower limb ischemia in a young woman with no known atherosclerotic history. The thrombus was successfully surgically removed, and the infective endocarditis was treated conservatively with satisfactory clinical and laboratory results. We opted for mitral valve repair surgery as a first-line treatment, given that the patient was of childbearing age, to avoid long-term anticoagulation.

In general, in cases of infective endocarditis, fever and systemic signs such as chills, asthenia, and weight loss are the most frequent symptoms. A new heart murmur develops in 48% of patients [7]. Biological signs of inflammation, such as elevated C-reactive protein, leukocytosis, and microcytic anemia, may be present but are not specific [8]. Vascular complications are also common [9]. However, cases of patients presenting with emboli in peripheral limb arteries are unusual [10]. Most studies report an incidence of 4% to 5% in patients with infective endocarditis of native heart valves [11].

The pathogen identified in our patient was *Staphylococcus intermedius*. Coagulase-positive infective endocarditis other than *Staphylococcus aureus* is rare [12].

Regarding imaging, the sensitivity of transthoracic echocardiography in infective endocarditis ranges from 21% to 58%, and that of transesophageal echocardiography ranges from 86% to 95% [13]. Echocardiographic findings are key criteria and may include, among others, the presence of vegetations, abscesses, and the development of a prosthetic valve dehiscence. In our case, transthoracic echocardiography revealed vegetations on the anterior mitral valve leaflet, which are likely the cause of the peripheral embolism.

The general approach to treating infective endocarditis involves stabilizing the patient's clinical condition, initiating antibiotic therapy according to the specific pathogen, and performing successive blood cultures to assess treatment efficacy. In our patient, empirical treatment was replaced with vancomycin and gentamicin based on the results of the antibiogram. European guidelines recommend penicillin as first-line treatment for infective endocarditis caused by methicillin-susceptible staphylococci [4].

Valve surgery is indicated in most patients with staphylococcal infective endocarditis and/or endocarditis associated with large vegetations exceeding 10 mm, or with one or more episodes of peripheral embolization [4]. In our patient, the small vegetation size, the single embolic episode, and the favorable response to antibiotic therapy supported a conservative approach.

Our case illustrates an unusual clinical presentation of infective endocarditis on a native valve and describes a delayed conservative surgical management of the native valve with a good outcome.

## Conclusions

Infective endocarditis is not a uniform disease due to its highly variable presentations, depending on the initial clinical manifestations, pre-existing heart disease, the causative microorganism, and the presence or absence of complications. An initial assessment, performed urgently, must evaluate the severity of complications, particularly embolic ones, which is a vital emergency, and trigger appropriate and effective therapeutic management as quickly as possible. This clinical case highlights the importance of suspecting infective endocarditis in cases of lower limb embolic events and encourages a conservative endocarditic surgical approach to the native valve.
